# The Psychological Impact of Dental Aesthetics in Patients with Juvenile Idiopathic Arthritis Compared with Healthy Peers: A Cross-Sectional Study

**DOI:** 10.3390/dj7040098

**Published:** 2019-10-01

**Authors:** Rosaria Bucci, Roberto Rongo, Alessandra Amato, Stefano Martina, Vincenzo D’Antò, Rosa Valletta

**Affiliations:** Department of Neurosciences, Reproductive Science and Oral Sciences, Division of Orthodontics, University of Naples Federico II, 80131 Naples, Italy; roberto.rongo@unina.it (R.R.); aale.amato@gmail.com (A.A.); stefano.martina@unina.it (S.M.); vincenzodanto@gmail.com (V.D.); valletta@unina.it (R.V.)

**Keywords:** quality of life, oral-health related quality of life, adolescent, juvenile idiopathic arthritis, dental aesthetics

## Abstract

This study aimed to assess whether dental aesthetics had a different impact on the psychosocial domains of adolescents with juvenile idiopathic arthritis (JIA) as compared with healthy peers. Fifty JIA patients and eighty controls aged between 13 and 17 years were enrolled. The Psychosocial Impact of Dental Aesthetics Questionnaire (PIDAQ) was administered along with tools for the self-assessment of malocclusion and self-esteem. An objective evaluation of malocclusion severity was performed through a clinical evaluation with the Dental Aesthetic Index (DAI). The sample was divided according to the DAI stages of malocclusion severity; a two-way analysis of variance (ANOVA) was performed to assess whether there was a difference in the studied variables according to the malocclusion and the presence of JIA. The results showed no interaction between the malocclusion severity and the presence of JIA in all analyzed variables (all *p* > 0.05). According to the DAI stages, the Dental Self-Confidence domain of the PIDAQ and the Perception of Occlusion Scale showed statistically significant differences only within the controls (*p* = 0.027 and *p* = 0.014, respectively). Therefore, JIA adolescents seem to be less concerned about their dental aesthetics compared with healthy peers, and clinicians should take particular care when proposing orthodontic treatments aiming only to improve dental aesthetics.

## 1. Introduction

Juvenile idiopathic arthritis (JIA) is a chronic form of rheumatic disease of unknown etiology that emerges before the age of 16 years and persists for a minimum of 6 weeks [1,2]. This broad term embraces numerous categories of clinically heterogeneous conditions that differ in terms of signs, symptoms, and the number of joints affected, as classified by the International League of Associations for Rheumatology (ILAR) [1]. Although great variability in the prevalence of JIA has been observed across the literature, this disease is reported amongst the most common chronic inflammatory rheumatic pathologies in children and adolescents [3]. Frequent clinical findings in patients with JIA are pain, fatigue, morning stiffness, and limited joint mobility, thus leading to impaired physical functions and compromising psychosocial domains [4].

Among the synovial joints affected by this chronic disease, the temporomandibular joint (TMJ) can also be involved, causing substantial jaw dysfunctions in daily oral activities [5]. The TMJ can be the first joint affected both uni- and bi-laterally, or it may be affected during the course of JIA [6]. Since the TMJ is often affected without clinically detectable signs and symptoms, the early detection of TMJ involvement and the assessment of TMJ damage is complex and requires a combination of clinical and radiological findings [7]. Depending on the JIA subtypes, the diagnostic criteria adopted, and the differences in disease duration, the prevalence of TMJ involvement has been estimated at between 30% and 87% [5,7]. The involvement of the TMJ in JIA adolescents might result in growth disturbances of the mandibular condyle, leading to craniofacial alternations such as posterior rotation of the mandible, mandibular retrognathia, micrognathia, and facial asymmetry [8,9]. In turn, these craniofacial alterations result in frequent dentoalveolar findings, such as Class II division 1, crossbite, and anterior open bite [10]. Hence, children with JIA are often candidates for functional or orthodontic treatments to correct Class II malocclusions, cross-bite, and open bite malocclusion according to their skeletal maturation [11,12,13].

To better understand the perception and the impact of a disease from the patient’s perspective, patient-reported outcome measures have been largely introduced in the clinical practice [14]. In this context, the Health-Related Quality of Life (HRQoL) assessment, by means of self-measuring standardized and validated questionnaires, provides important information regarding how individuals feel about certain aspects of their lives with respect to their health or health condition, considering physical, mental, and social health dimensions [15]. Previous studies have reported that adolescents with JIA present impaired HRQoL as compared with those of healthy peers, particularly concerning physical symptoms [16,17]. Determinants that have been identified for impaired HRQoL include polyarticular arthritis or extended oligoarticular arthritis, short disease duration, pain, disabilities, and increased disease severity [18]. Interestingly, in a significant percentage of children and adolescents affected by JIA, the HRQoL seems to be suboptimal also when mild or no clinical symptoms are present [19]. Whenever the TMJ is involved and JIA-induced orofacial symptoms are present, comfort while eating and/or sleeping and/or engaging in social interaction is disturbed, thus affecting the domains of the Oral Health-Related Quality of Life (OHRQoL) [20].

Dental malocclusion is one of the most prevalent oral conditions in children and adolescents, along with dental caries and periodontal disease [21,22,23]. Recent systematic reviews have pointed out that malocclusions have a negative impact on OHRQoL, and the more severe the malocclusion, the worse the impact is on some physical and psychosocial domains [24,25]. However, the subjective perception of malocclusion and need for orthodontic treatment substantially differs from the objective perspective of the clinician [26]. The differences in the self-perceived dentofacial aesthetics are due to subjective considerations, self-esteem, gender, age, and socio-economic background [27]. In 2006, Klages and co-workers developed a specific multi-item questionnaire to measure the impact of dental aesthetics on quality of life (Psychosocial Impact of Dental Aesthetics Questionnaire, PIDAQ) [28]. This tool was originally developed for young adults (18–30 years of age), but was later adapted and modified for its use among adolescents [29]. Due to its good psychometric properties and ease of use, the PIDAQ has been translated and validated in numerous languages [30,31,32].

The evaluation of the impact of dental aesthetics on quality of life in children and adolescents is crucially important since the subjective perception of treatment need could affect motivation and compliance during the orthodontic therapy [33]. Furthermore, it has been largely proven that dental aesthetics has a primary role in impaired social interactions with peers and bullying [34,35]. This can be even more important when dealing with adolescents with suboptimal quality of life, such as JIA patients [14]. Indeed, these children should be treated with physical therapy, psychological support, and the fostering of good integration with peers in order to promote adjustment and provide coping skills to deal with their non-curable chronic pathology [36]. However, no studies provided information on the psychosocial impact of perceived dental aesthetics in JIA patients. Therefore, the aim of the current cross-sectional survey was to assess the impact of dental aesthetics on the psychosocial domains of adolescents affected by a systemic pathology involving the stomatognathic system (JIA) as compared with that of healthy peers. 

## 2. Materials and Methods

### 2.1. Sample

**Figure 1:**
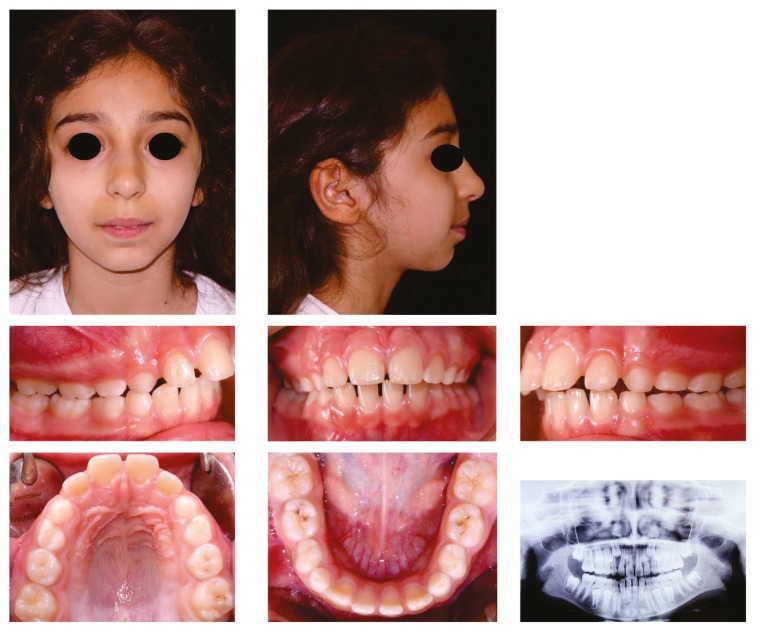
Full orthodontic records of one female patient affected by juvenile idiopathic arthritis (JIA). Extraoral and intraoral pictures show a concave profile and bilateral Angle Class II relationship. The panoramic radiograph suggests advanced degeneration of left and right condyles.

The study was approved by the local ethics committee of the University of Naples Federico II (protocol number 169/18, 15/05/2018). A consecutive sample of adolescents with a diagnosis of JIA (Figure 1), as defined by the ILAR criteria [1], was recruited among the patients attending the clinic of Pediatric Rheumatology of the University of Naples Federico II (Naples, Italy). Healthy controls free from JIA were recruited among the individuals attending the clinic of dentistry at the University of Naples Federico II (Naples, Italy) for a first consultation. The recruitment of both groups was performed between May 2018 and October 2018. In accordance with the Declaration of Helsinki, written informed consent was obtained from the parents of the participants, who were informed of the aim and study procedures. The inclusion criteria were the following: age between 13 and 17 years, diagnosis of JIA (only for the JIA group), and the willingness to participate in the study. Genetic syndromes, orofacial abnormalities, intellectual and/or physical inability to answer the questionnaires, previous orthodontic treatment, the presence of cavities, missing or fractured teeth, and dark areas on the frontal teeth were considered exclusion criteria since they could influence the self-assessment of the malocclusion and of the dental aesthetics. 

All subjects were invited to fill in written questionnaires to assess their psychological status. This was done with the help of their parents and the constant supervision of a clinician to ensure the correct comprehension of the questionnaires. Furthermore, a clinical examination was performed by one calibrated operator to assess the objective degree of malocclusion.

### 2.2. Data Collection

- Psychosocial Impact of Dental Aesthetics Questionnaire (PIDAQ): The Italian translated version of the PIDAQ, adapted for its use among adolescents, was used [37]. The questionnaire is composed of 23 items distributed among three subscales: Aesthetic Concern (AC, 4 items), Psychosocial Impact (PSI, 13 items), and Dental Self-Confidence (DSC, 6 items). Each item is scored on a five-point scale with the following response options: “not at all” = 0; “a little” = 1; “somewhat” = 2; “strongly” = 3; and “very strongly” = 4. For PSI and AC, a score of 0 indicates no impact of dental aesthetics on OHRQoL while a score of 4 indicates maximum impact. Only the items of the DSC show positive meaning and reverse interpretation [28].
- Perception of Occlusion Scale (POS): The POS is a tool to self-assess the arrangement of the anterior teeth and it comprises 6 items referring to upper and lower crowding and irregularity, spacing between upper incisors, and open bite. A 4-point answering format was presented with “not at all” = 1; “a little” = 2; “moderate” = 3; and “strong” = 4 [38].
- Aesthetic Component of the Index of Orthodontic Treatment Need (AC-IOTN): The AC-IOTN is composed of 10 photographs of the front teeth displaying increasing severity of malocclusion. The individuals were asked to indicate which photograph (1 to 10) they thought most closely resembled their own dentition [39].
- Rosenberg Self-Esteem Scale: The Rosenberg Self-Esteem Scale is a 10-item scale that determines global self-worth by measuring both positive and negative feelings about the self. All items are answered using a 4-point Likert scale format ranging from “strongly agree” to “strongly disagree”. The first five statements are formulated in a positive form, with the remaining five in a negative form [40].
- Dental Aesthetic Index (DAI): DAI evaluates 10 occlusal characteristics: overjet, mandibular overjet, tooth loss, diastema, anterior open bite, anterior crowding, anterior diastema, the largest mandibular anterior irregularities, the largest maxillary anterior irregularities and sagittal molar relationship [41]. The DAI presents four stages of malocclusion severity: a score lower than or equal to 25 (no or slight treatment need), a score between 26 and 30 (elective treatment), a score between 31 and 35 (treatment highly desirable), and a score greater than or equal to 36 (treatment mandatory).

### 2.3. Sample Size

In a two-way ANOVA study with numerator dF (degree of freedom) of 3, a total sample size of 126 was obtained from the groups whose means are to be compared. Considering a medium effect size of 0.3, this sample size achieves 80% power to detect differences among the means versus the alternative of equal means using an F test with a significance level *p* < 0.05. 

### 2.4. Statistical Analysis

Descriptive statistics was performed with regard to age, gender, arthritis diagnosis, pharmacological treatment, and all the analyzed questionnaires. The Shapiro–Wilk test was performed to assess the distribution of the data. Continuous data were reported as means ± standard deviation (SD); nominal data were reported as frequencies. The sample was divided into four subgroups according to the DAI stages of malocclusion severity, and a two-way analysis of variance (ANOVA) was used to evaluate whether there was a difference in the assessed variables according to the severity of malocclusion and the presence of JIA. The statistical models were also adjusted for age and gender. Statistical Package for Social Science (SPSS) version 22.0 for Windows (SPSS IBM, Armonk, NY, USA) was used to perform the statistical analysis. The significance level was set at *p* < 0.05.

## 3. Results

### 3.1. Sample Characteristics

The sample comprised 130 subjects: 50 subjects with JIA (20 boys, 30 girls, mean age 15.0 ± 1.7, JIA group) and 80 controls (30 boys, 50 girls, mean age 15.1 ± 1.6, Control group). The total sample presented a mean age of 15.0 ± 1.6 years. 

Of the 50 JIA patients, 31 individuals presented a diagnosis of oligoarticular arthritis, 18 individuals presented a diagnosis of polyarticular arthritis, and 1 individual presented a diagnosis of systemic arthritis.

Regarding the therapy, 17 patients were in treatment with methotrexate (Reumaflex), 13 patients were in treatment with biologic medications (Embrel, RoActemra), 7 patients were in treatment with nonsteroidal anti-inflammatory drugs (naproxen), and 13 were not undergoing any therapies.

### 3.2. Data Collection

In the JIA group, 17 adolescents presented a DAI index equal to or lower than 25, 13 adolescents between 25 and 30, 7 adolescents between 31 and 35, and 13 adolescents equal to or greater than 36. In the Control group, 32 subjects presented a DAI index equal to or lower than 25, 13 subjects between 25 and 30, 13 subjects between 31 and 35, and 22 subjects equal to or greater than 36. Moreover, the mean value of the total DAI did not differ significantly between the two groups (JIA: 30.34 ± 9.49 vs. Control: 29.20 ± 8.88, *p* = 0.489, Table 1). 

The mean values for the analyses variables are reported in Table 1. When comparing the scores between subjects with and without JIA, it was noted that only three variables showed statistically significant differences: DSC (*p* = 0.013, JIA: 10.18 ± 6.35 vs. Control: 7.67 ± 5.69); POS (*p* = 0.017, JIA: 3.26 ± 3.13 vs. Control: 4.66 ± 3.99); and Rosenberg (*p* = 0.046, JIA: 20.24 ± 1.74 vs. Control: 19.54 ± 1.83).

The two-way ANOVA did not show any significant difference when analyzing the interaction between the effect of malocclusion severity and presence of JIA (all *p* > 0.05; Table 2). 

According to the four stages of malocclusion severity as assessed with DAI, the DSC domain of the PIDAQ (*p* = 0.027) and the POS (*p* = 0.014) showed a statistically significant difference only within the Control group (Table 2). Furthermore, statistically significant difference was observed in both groups for the AC-IOTN (JIA: *p* = 0.002; Control: *p* = 0.006, Table 2).

Both statistical models, adjusted for age and gender, confirmed the achieved results (Table 1 and Table 2).

## 4. Discussion

This cross-sectional survey investigates whether the existence of a systemic disease affecting the stomatognathic system, such as JIA, modifies the psychosocial impact of the perception of dental aesthetics in adolescents. The objective malocclusion was determined by means of clinical examination and scored by means of the DAI, while questionnaires were used to measure five different self-assessed aspects (DSC, AC, PSI, AC-IOTN, and POS) of dental aesthetics.

It has been largely proven that the assessment of HRQoL in children with chronic disease is crucially important [42], and several generic or disease-specific tools are available in the literature to measure these outcomes [43,44]. In the dental field, the OHRQoL is the result of a complex interaction of psychological, cultural, physical, and social aspects, and its evaluation is fundamental for measuring the real benefit of a medical therapy [44]. Therefore, since orthodontic treatment is usually a long-term therapy that can decrease the OHRQoL during its active course [45], the choice to start orthodontic therapy in adolescents that already present poor quality of life must be carefully planned. Furthermore, as supported by the Minorities’ Diminished Returns theory [46], an improvement of oral health or the correction of a malocclusion in a disadvantaged population might not be associated with an improvement of the psychological well-being.

In the between-group comparisons, DSC and POS resulted statistically significant different suggesting that the JIA patients presented less concern for their occlusion and for the aesthetic of their teeth. Furthermore, the difference in the Rosenberg score supported that JIA adolescents presented slightly higher self-esteem when compared with the healthy controls. One previous study performed on a multi-ethnic cohort of 10-year-old children reported that self-esteem modifies the relationship between subjective orthodontic treatment need and the OHRQoL [47]. Interestingly, in the current sample the severity of malocclusion was similar between the two groups as reported with the DAI scores, and subjects belonging to both groups were correctly able to identify their own severity of malocclusion, as shown with the AC-IOTN scores. Hence, the results of the current study support that adolescents affected by a chronic rheumatic disease might be less focused on their own dental aspects, showing better emotional state related to their own evaluation of the dental appearance. On the other hand, the control group of adolescents without JIA was consecutively recruited among individuals attending the orthodontic clinic for a first consultation. Therefore, it might be speculated that those subjects and their parents who were seeking orthodontic treatment were more concerned about their dental status and the appearance of their teeth [48]. Hence, these findings might not be extended to the general population of healthy adolescents. 

The two-way ANOVA showed no interaction between the severity of the malocclusion and the presence of the systematic disease. Indeed, for each stage of malocclusion severity, the mean values of the assessed variables in the JIA and the control group did not present any statistically significant difference. However, within the individual groups studied, as the severity of objective malocclusion increased, two variables showed different behaviors in the control group when compared with the JIA group. In particular, only subjects belonging to the control group presented significantly different values of DSC and POS as the severity of the malocclusion increased, whereas similar behavior was observed for the AC-IOTN in the two groups. These findings further support that patients affected by a systemic pathology might be less concerned about the malocclusion, unlike healthy patients.

A possible explanation for less attention paid by adolescents with JIA to their dental aesthetic could be due to some aspects related to their daily lives. Indeed, JIA patients usually present a lower physical well-being dimension associated with increased difficulties in performing regular physical activity as compared with healthy subjects [49,50], and this was highly correlated with a lower HRQoL among JIA adolescents [51]. Moreover, these patients are constantly treated for their chronic pathology and they also undergo regular medical consultations due to pain, physical disability and eye-related problems (uveitis) that negatively affect their emotions, their possibility to attend school and their daily activities [52]. Finally, since the progression of the pathology is characterized by frequent relapses, these patients require constant follow-up over the years, which is associated with a high number of hospitalizations, stressful treatment experiences (e.g., repeated intravenous infusions, frequent injections), and regular multi-professional treatment approaches [53]. Hence, due to the very complex medical context that begin in early childhood, it can be speculated that lower awareness is related to the aesthetic aspects of the teeth. However, recent studies showed that HRQoL in JIA patients improved gradually over time, and particular improvement has been observed in the recent years thanks to the increased efficacy and the increased handling of the new drugs [54]. Indeed, whenever treatment with non-steroidal anti-inflammatory drugs, intra-articular corticosteroid injections, or methotrexate or sulfasalazine is not sufficient to provide adequate control of the pathology, the shift to biological drugs is recommended to improve the therapy outcomes [54]. For instance, in a large longitudinal cohort study with a long-term follow-up of 34 months, only 8–14% of JIA patients presented alarming trajectories of persistently poor HRQoL, and high initial levels of disease activity seemed to be predictive factors of an unfavorable trajectory [55].

Patient-centered outcome research is commonly approached with the use of questionnaires that allow the self-measurement of a number of factors that contribute to an individual’s wellbeing, with particular attention to concerns prioritized by patients [14]. The PIDAQ is a specific orthodontic-related tool promoted to measure the psychosocial impact of dental aesthetic characteristics, without considering oral functions and pain. The translated Italian version of the PIDAQ showed good psychometric characteristics among Italian adolescents, allowing for the identification of small changes in adolescents’ quality of life [37]. Notwithstanding, since the self-assessment of a malocclusion with POS and AC-IOTN can be influenced by several psychological and social factors, an additional clinical measurement (DAI) was introduced in the current study to objectively rank the severity of the malocclusion according to the level of treatment need and the amount of deviation from normal occlusion [41].

In children with chronic disease parental education, occupation, marital status, income and health insurance coverage play a crucial role in the QoL. In particular, it has been observed that children from lower socio-economic backgrounds presented reduced QoL compared with their wealthier peers [56]. However, in the current survey socio-economic status was not collected, thus presenting a major limitation of the study. Furthermore, the cross-sectional design of the study did not consider whether the fluctuation of the disease activity could influence the perception of dental aesthetics in JIA patients. This is the first study that evaluated the perception of dental aesthetics in adolescents with systemic diseases involving the stomatognathic system with a specific cross-culturally adapted and validated questionnaire.

## 5. Conclusions

The dental aesthetics in adolescents with JIA did not significantly alter their psychosocial domains as much as it did for healthy peers. Therefore, orthodontic treatment aiming only to improve the dental aesthetics of JIA patients must be done with caution since no clear impairment of the psychological and social lives of these individuals has been observed due to the dental malocclusion. 

## Figures and Tables

**Table 1:** Descriptive statistics and results of the univariate test of the two-way analysis of variance (ANOVA) between juvenile idiopathic arthritis (JIA) subjects and controls. *p*-value adjusted for age and gender are reported in italics. Statistically significant differences (*p* < 0.05) are reported in bold text.

Variable	Group	Mean	SD	95% CI		*p*-Value
				Lower Limit	Upper Limit	
AC	JIA	4.20	3.29	3.26	5.13	0.191
	Control	4.96	3.14	4.26	5.66	0.226
PSI	JIA	11.34	11.46	8.08	14.60	0.841
	Control	10.44	9.72	8.27	12.60	0.735
DSC	JIA	10.18	6.35	8.38	11.98	0.013
	Control	7.67	5.69	6.41	8.94	0.015
POS	JIA	3.26	3.13	2.37	4.15	0.017
	Control	4.66	3.99	3.78	5.55	0.019
AC-IOTN	JIA	2.90	2.04	2.32	3.48	0.719
	Control	2.98	1.83	2.57	3.38	0.777
Rosenberg	JIA	20.24	1.74	19.74	20.74	0.046
	Control	19.54	1.83	19.13	19.95	0.043
DAI	JIA	30.34	9.49	27.64	33.04	0.489
	Control	29.20	8.88	27.22	31.18	0.489

AC: Aesthetic Component; PSI: Psychosocial Impact; DSC: Dental Self-Confidence; POS: Perception of Occlusion Scale; AC-IOTN: Aesthetic Component of the Index of Orthodontic Treatment Need; DAI: Dental Aesthetic Index; SD: Standard Deviation; CI: Confidence Interval.

**Table 2:** Results of the two-way analysis of variance (ANOVA) according to the four malocclusion severity stages and presence of juvenile idiopathic arthritis (JIA), and results of the univariate test for the malocclusion severity stages within each group. *p*-Values adjusted for age and gender are reported in italics. Statistically significant differences (*p* < 0.05) are reported in bold.

Variable	Group	DAI	Mean	SD	95% CI		*p*-Value Univariate Test	*p*-Value ANOVA
					Lower Limit	Upper Limit		
AC	JIA	≤25	3.76	3.19	2.12	5.41	0.723 0.616	0.932 0.932
		25–30	4.00	3.16	2.09	5.91		
		31–35	4.00	2.31	1.86	6.14		
		≥36	5.08	4.09	2.60	7.55		
	Control	≤25	4.66	2.60	3.720	5.593	0.914 0.866	
		25–30	5.00	3.89	2.647	7.353		
		31–35	5.23	2.31	3.832	6.630		
		≥36	5.23	3.89	3.502	6.952		
PSI	JIA	≤25	6.76	6.88	3.23	10.30	0.090 0.078	0.775 0.789
		25–30	13.00	14.47	4.26	21.74		
		31–35	10.57	10.10	1.23	19.91		
		≥36	16.08	11.32	9.23	22.92		
	Control	≤25	7.34	9.20	4.03	10.66	0.126 0.090	
		25–30	14.38	8.71	9.12	19.65		
		31–35	10.61	10.98	3.98	17.25		
		≥36	12.50	9.47	8.30	16.70		
DSC	JIA	≤25	12.12	7.14	8.45	15.79	0.313 0.255	0.932 0.957
		25–30	10.23	7.11	5.93	14.53		
		31–35	8.29	5.31	3.37	13.20		
		≥36	8.61	4.69	5.79	11.44		
	Control	≤25	9.97	7.11	7.40	12.53	0.027 0.018	
		25–30	6.77	4.38	4.12	9.42		
		31–35	4.69	3.15	2.79	6.59		
		≥36	6.64	3.90	4.91	8.36		
POS	JIA	≤25	2.12	3.43	0.36	3.88	0.207 0.237	0.428 0.531
		25–30	3.15	2.97	1.36	4.95		
		31–35	3.14	2.48	0.85	5.44		
		≥36	4.92	2.78	3.24	6.60		
	Control	≤25	3.41	3.69	2.08	4.74	0.014 0.008	
		25–30	4.08	3.23	2.13	6.03		
		31–35	7.00	4.38	4.35	9.65		
		≥36	5.45	4.02	3.67	7.24		
AC-IOTN	JIA	≤25	1.76	0.66	1.42	2.11	0.002 0.001	0.272 0.279
		25–30	2.62	1.44	1.74	3.49		
		31–35	3.86	2.91	1.16	6.55		
		≥36	4.15	2.44	2.68	5.63		
	Control	≤25	2.19	1.09	1.79	2.58	0.006 0.004	
		25–30	3.31	1.80	2.22	4.39		
		31–35	4.15	2.41	2.70	5.61		
		≥36	3.23	1.93	2.37	4.08		
Rosenberg	JIA	≤25	20.06	1.56	19.26	20.86	0.458 0.587	0.635 0.596
		25–30	20.69	2.06	19.45	21.93		
		31–35	19.43	2.07	17.51	21.34		
		≥36	20.46	1.45	19.58	21.34		
	Control	≤25	19.87	1.47	19.34	20.41	0.303 0.324	
		25–30	19.85	1.86	18.72	20.97		
		31–35	18.92	1.75	17.86	19.98		
		≥36	19.23	2.27	18.22	20.23		

AC: Aesthetic Component; PSI: Psychosocial Impact; DSC: Dental Self-Confidence; POS: Perception of Occlusion Scale; AC-IOTN: Aesthetic Component of the Index of Orthodontic Treatment Need; DAI: Dental Aesthetic Index; SD: Standard Deviation; CI: Confidence Interval.

## References

[1] Petty R.E., Southwood T.R., Manners P., Baum J., Glass D.N., Goldenberg J., He X., Maldonado-Cocco J., Orozco-Alcala J., Prieur A.-M. (2004). International League of Associations for Rheumatology classification of juvenile idiopathic arthritis: Second revision, Edmonton, 2001. J. Rheumatol..

[2] Ravelli A., Martini A. (2007). Juvenile idiopathic arthritis. Lancet (London, England).

[3] Thierry S., Fautrel B., Lemelle I., Guillemin F. (2014). Prevalence and incidence of juvenile idiopathic arthritis: A systematic review. Jt. Bone Spine.

[4] Hanns L., Cordingley L., Galloway J., Norton S., Carvalho L.A., Christie D., Sen D., Carrasco R., Rashid A., Foster H. (2018). Depressive symptoms, pain and disability for adolescent patients with juvenile idiopathic arthritis: Results from the Childhood Arthritis Prospective Study. Rheumatology.

[5] Carrasco R. (2015). Juvenile idiopathic arthritis overview and involvement of the temporomandibular joint: Prevalence, systemic therapy. Oral Maxillofac. Surg. Clin. N. Am..

[6] Ringold S., Cron R.Q. (2009). The temporomandibular joint in juvenile idiopathic arthritis: Frequently used and frequently arthritic. Pediatr. Rheumatol. Online J..

[7] Rongo R., Alstergren P., Ammendola L., Bucci R., Alessio M., D’Anto V., Michelotti A. (2019). Temporomandibular joint damage in juvenile idiopathic arthritis: Diagnostic validity of diagnostic criteria for temporomandibular disorders. J. Oral. Rehabil..

[8] Billiau A.D., Hu Y., Verdonck A., Carels C., Wouters C. (2007). Temporomandibular joint arthritis in juvenile idiopathic arthritis: Prevalence, clinical and radiological signs, and relation to dentofacial morphology. J. Rheumatol..

[9] Hsieh Y.-J., Darvann T.A., Hermann N.V., Larsen P., Liao Y.-F., Bjoern-Joergensen J., Kreiborg S. (2016). Facial morphology in children and adolescents with juvenile idiopathic arthritis and moderate to severe temporomandibular joint involvement. Am. J. Orthod. Dentofacial. Orthop..

[10] Chatzigianni A., Kyprianou C., Papadopoulos M.A., Sidiropoulou S. (2018). Dentoalveolar characteristics in children with juvenile idiopathic arthritis. J. Orofac. Orthop..

[11] D’Anto V., Bucci R., Franchi L., Rongo R., Michelotti A., Martina R. (2015). Class II functional orthopaedic treatment: A systematic review of systematic reviews. J. Oral. Rehabil..

[12] Rongo R., Valleta R., Bucci R., Bonetti G.A., Michelotti A., D’Antò V. (2015). Does clinical experience affect the reproducibility of cervical vertebrae maturation method?. Angle Orthod..

[13] Michelotti A., Rongo R., Valentino R., D’Anto V., Bucci R., Danzi G., Cioffi I. (2019). Evaluation of masticatory muscle activity in patients with unilateral posterior crossbite before and after rapid maxillary expansion. Eur. J. Orthod..

[14] Weitzman E.R., Wisk L.E., Salimian P.K., Magane K.M., Dedeoglu F., Hersh A.O., Kimura Y., Mandl K.D., Ringold S., Natter M. (2018). Adding patient-reported outcomes to a multisite registry to quantify quality of life and experiences of disease and treatment for youth with juvenile idiopathic arthritis. J. Patient Rep. Outcomes.

[15] World Health Organization The Structure of the WHOQOL-100. WHOQOL: Measuring Quality of Life. https://www.who.int/healthinfo/survey/whoqol-qualityoflife/en/..

[16] Gutierrez-Suarez R., Pistorio A., Cespedes Cruz A., Gutierrez-Suarez R., Pistorio A., Cespedes Cruz A., Norambuena X., Flato B., Rumba I., Huemer C. (2007). Health-related quality of life of patients with juvenile idiopathic arthritis coming from 3 different geographic areas. The PRINTO multinational quality of life cohort study. Rheumatology (Oxford).

[17] Oliveira S., Ravelli A., Pistorio A., Castell E., Malattia C., Prieur A.M., Foeldvari I. (2007). Proxy-reported health-related quality of life of patients with juvenile idiopathic arthritis: The Pediatric Rheumatology International Trials Organization multinational quality of life cohort study. Arthritis Rheum..

[18] Abdul-Sattar A.B., Elewa E.A., El-Shahawy E.E.-D., Waly E.H. (2014). Determinants of health-related quality of life impairment in Egyptian children and adolescents with juvenile idiopathic arthritis: *Sharkia Gov*. Rheumatol. Int..

[19] Seid M., Opipari L., Huang B., Brunner H.I., Lovell D.J. (2009). Disease control and health-related quality of life in juvenile idiopathic arthritis. Arthritis Rheum..

[20] Rahimi H., Twilt M., Herlin T., Spiegel L., Pedersen T.K., Küseler A., Stoustrup P. (2018). Orofacial symptoms and oral health-related quality of life in juvenile idiopathic arthritis: A two-year prospective observational study. Pediatr. Rheumatol. Online J..

[21] Paduano S., Rongo R., Bucci R., Aiello D., Carvelli G., Ingenito A., Cantile T., Ferrazzano G.F. (2018). Is there an association between various aspects of oral health in Southern Italy children? An epidemiological study assessing dental decays, periodontal status, malocclusions and temporomandibular joint function. Eur. J. Paediatr. Dent..

[22] Perrotta S., Bucci R., Simeon V., Martina S., Michelotti A., Valletta R. (2019). Prevalence of malocclusion, oral parafunctions and temporomandibular disorder-pain in Italian schoolchildren: An epidemiological study. J. Oral Rehabil..

[23] Paduano S., Bucci R., Rongo R., Silva R., Michelotti A. (2018). Prevalence of temporomandibular disorders and oral parafunctions in adolescents from public schools in Southern Italy. Cranio.

[24] Kragt L., Dhamo B., Wolvius E.B., Ongkosuwito E.M. (2016). The impact of malocclusions on oral health-related quality of life in children-a systematic review and meta-analysis. Clin. Oral. Investig..

[25] Sun L., Wong H.M., McGrath C.P. (2017). Relationship Between the Severity of Malocclusion and Oral Health Related Quality of Life: A Systematic Review and Meta-analysis. Oral Health Prev. Dent..

[26] Twigge E., Roberts R.M., Jamieson L., Dreyer C.W., Sampson W.J. (2016). Qualitative evaluation of pretreatment patient concerns in orthodontics. Am. J. Orthod. Dentofacial. Orthop..

[27] Peres K.G., Barros A.J.D., Anselmi L., Peres M.A., Barros F.C. (2008). Does malocclusion influence the adolescent’s satisfaction with appearance? A cross-sectional study nested in a Brazilian birth cohort. Commun. Dent. Oral Epidemiol..

[28] Klages U., Claus N., Wehrbein H., Zentner A. (2006). Development of a questionnaire for assessment of the psychosocial impact of dental aesthetics in young adults. Eur. J. Orthod..

[29] Klages U., Erbe C., Sandru S.D., Brullman D., Wehrbein H. (2015). Psychosocial impact of dental aesthetics in adolescence: Validity and reliability of a questionnaire across age-groups. Qual Life Res..

[30] Sardenberg F., Oliveira A.C., Paiva S.M., Auad S.M., Vale M.P. (2011). Validity and reliability of the Brazilian version of the psychosocial impact of dental aesthetics questionnaire. Eur. J. Orthod..

[31] Spalj S., Lajnert V., Ivankovic L. (2014). The psychosocial impact of dental aesthetics questionnaire--translation and cross-cultural validation in Croatia. Qual. Life Res..

[32] Bucci R., Rongo R., Zito E., Galeotti A., Valletta R., D’Anto V. (2015). Cross-cultural adaptation and validation of the Italian Psychosocial Impact of Dental Aesthetics Questionnaire (PIDAQ). Qual. Life Res..

[33] El-Huni A., Colonio Salazar F.B., Sharma P.K., Fleming P.S. (2019). Understanding factors influencing compliance with removable functional appliances: A qualitative study. Am. J. Orthod. Dentofacial. Orthop..

[34] Scheffel D.L.S., Jeremias F., Fragelli C.M.B., Dos Santos-Pinto L.A.M., Hebling J., de Oliveira O.B.J. (2014). Esthetic dental anomalies as motive for bullying in schoolchildren. Eur. J. Dent..

[35] Chan A., Antoun J.S., Morgaine K.C., Farella M. (2017). Accounts of bullying on Twitter in relation to dentofacial features and orthodontic treatment. J. Oral. Rehabil..

[36] Tong A., Jones J., Craig J.C., Singh-Grewal D. (2012). Children’s experiences of living with juvenile idiopathic arthritis: A thematic synthesis of qualitative studies. Arthritis Care Res (Hoboken).

[37] Bucci R., Rongo R., Zito E., Valletta R., Michelotti A., D’anto V. (2017). Translation and validation of the italian version of the Psychosocial Impact of Dental Aesthetics Questionnaire (pidaq) among adolescents. Eur. J. Paediatr. Dent..

[38] Espeland L.V., Stenvik A. (1991). Perception of personal dental appearance in young adults: Relationship between occlusion, awareness, and satisfaction. Am. J. Orthod. Dentofacial. Orthop..

[39] Brook P.H., Shaw W.C. (1989). The development of an index of orthodontic treatment priority. Eur. J. Orthod..

[40] Rosenberg M. (1965). Society and the Adolescent Self-Image.

[41] Cons N.C., Jenny J., Kohout F.J., Songpaisan Y., Jotikastira D. (1989). Utility of the dental aesthetic index in industrialized and developing countries. J. Public Health Dent..

[42] Eiser C., Jenney M. (2007). Measuring quality of life. Arch. Dis. Child.

[43] Angeles-Han S.T., Yeh S., McCracken C., Jenkins K., Stryker D., Myoung E., Vogler L.B., Rouster-Stevens K., Lambert S.R., Harrison M.J. (2015). Using the Effects of Youngsters’ Eyesight on Quality of Life Questionnaire to Measure Visual Outcomes in Children With Uveitis. Arthritis Care Res. (Hoboken).

[44] Tadic V., Cooper A., Cumberland P., Lewando-Hundt G., Rahi J.S. (2016). Measuring the Quality of Life of Visually Impaired Children: First Stage Psychometric Evaluation of the Novel VQoL_CYP Instrument. PLoS ONE.

[45] Choi S.-H., Cha J.-Y., Lee K.-J., Yu H.-S., Hwang C.-J. (2017). Changes in psychological health, subjective food intake ability and oral health-related quality of life during orthodontic treatment. J. Oral. Rehabil..

[46] Assari S. (2017). Unequal Gain of Equal Resources across Racial Groups. Int. J. Heal Policy Manag..

[47] Kragt L., Wolvius E.B., Jaddoe V.W.V., Tiemeier H., Ongkosuwito E.M. (2018). Influence of self-esteem on perceived orthodontic treatment need and oral health-related quality of life in children: The Generation R Study. Eur. J. Orthod..

[48] Lin F., Ren M., Yao L., He Y., Guo J., Ye Q. (2016). Psychosocial impact of dental esthetics regulates motivation to seek orthodontic treatment. Am. J. Orthod. Dentofacial Orthop..

[49] Shaw K.L., Southwood T.R., Duffy C.M., McDonagh J.E. (2006). Health-related quality of life in adolescents with juvenile idiopathic arthritis. Arthritis Rheum..

[50] Manczak M., Rutkowska-Sak L., Raciborski F. (2016). Health-related quality of life in children with juvenile idiopathic arthritis—Child’s and parent’s point of view. Reumatologia.

[51] Kwon H.-J., Kim Y.L., Lee S.M. (2015). Relation between functional ability and health-related quality of life of children with juvenile rheumatoid arthritis. J. Phys. Ther. Sci..

[52] Sen E.S., Morgan M.J., MacLeod R., Strike H., Hinchcliffe A., Dick A.D., Muthusamy B., Ramanan A.V. (2017). Cross sectional, qualitative thematic analysis of patient perspectives of disease impact in juvenile idiopathic arthritis-associated uveitis. Pediatr. Rheumatol. Online J..

[53] Cavazzana L., Fornili M., Filocamo G., Agostoni C., Auxilia F., Castaldi S. (2018). Hospital clinical pathways for children affected by juvenile idiopathic arthritis. Ital. J. Pediatr..

[54] Listing M., Monkemoller K., Liedmann I., Niewerth M., Sengler C., Listing J., Foell D., Heiligenhaus A., Klein A., Horneff G. (2018). The majority of patients with newly diagnosed juvenile idiopathic arthritis achieve a health-related quality of life that is similar to that of healthy peers: Results of the German multicenter inception cohort (ICON). Arthritis Res. Ther..

[55] Oen K., Guzman J., Dufault B., Tucker L.B., Shiff N.J., Duffy K.W., Huber A.M. (2018). Health-Related Quality of Life in an Inception Cohort of Children With Juvenile Idiopathic Arthritis: A Longitudinal Analysis. Arthritis Care. Res. (Hoboken).

[56] Didsbury M.S., Kim S., Medway M.M., Tong A., McTaggart S.J., Walker A.M., White S., Mackie F.E., Kara T., Craig J.C. (2016). Socio-economic status and quality of life in children with chronic disease: A systematic review. J. Paediatr. Child Health.

